# Intradermal grass pollen immunotherapy increases T_H_2 and IgE responses and worsens respiratory allergic symptoms

**DOI:** 10.1016/j.jaci.2016.09.024

**Published:** 2017-06

**Authors:** Anna Slovick, Abdel Douiri, Rachel Muir, Andrea Guerra, Konstantinos Tsioulos, Evie Hay, Emily P.S. Lam, Joanna Kelly, Janet L. Peacock, Sun Ying, Mohamed H. Shamji, David J. Cousins, Stephen R. Durham, Stephen J. Till

**Affiliations:** a Division of Asthma, Allergy and Lung Biology, King's College London, School of Medicine, Guy's Hospital, London, United Kingdom; b Division of Health and Social Care Research, King's College London, 4th floor Addison House, Guy's Campus, London, United Kingdom; c Clinical Research Facility, NIHR Biomedical Research Centre, Guy's Hospital, London, United Kingdom; d King's Clinical Trials Unit, King's College London, Institute of Psychiatry, London, United Kingdom; e Allergy and Clinical Immunology, National Heart and Lung Institute, Faculty of Medicine, Imperial College, London, United Kingdom; f Department of Infection, Immunity and Inflammation, NIHR Leicester Respiratory Biomedical Research Unit, Leicester Institute for Lung Health, University of Leicester, Leicester, United Kingdom; g MRC-Asthma UK Centre for Allergic Mechanisms of Asthma, London, United Kingdom

**Keywords:** Allergy immunotherapy, allergic rhinitis, grass pollen, *Phleum pratense*, immunotherapy, intradermal, low dose, APAAP, Alkaline phosphatase–anti-alkaline phosphatase, ARIA, Allergic Rhinitis and Its Impact on Asthma, AUC, Area under the curve, BU, Biological units, CRTH2, Chemoattractant receptor-homologous molecule expressed on T_H_2 lymphocytes, CXCR3, Chemokine (C-X-C motif) receptor 3, DC, Dendritic cell, IQR, Interquartile range, Mini-RQLQ, Mini-Rhinoconjunctivitis Quality of Life Questionnaire, PE, Phycoerythrin, PollenLITE, Pollen Low Dose Intradermal Therapy Evaluation, VAS, Visual analog scale, WAO, World Allergy Organization

## Abstract

**Background:**

Repeated low-dose grass pollen intradermal allergen injection suppresses allergen-induced cutaneous late-phase responses comparably with conventional subcutaneous and sublingual immunotherapy.

**Objective:**

We sought to evaluate the efficacy and safety of grass pollen intradermal immunotherapy in the treatment of allergic rhinitis.

**Methods:**

We randomly assigned 93 adults with grass pollen–induced allergic rhinitis to receive 7 preseasonal intradermal allergen injections (containing 7 ng of Phl p 5 major allergen) or a histamine control. The primary end point was daily combined symptom-medication scores during the 2013 pollen season (area under the curve). Analysis was by intention to treat. Skin biopsy specimens were collected after intradermal allergen challenges, and late-phase responses were measured 4 and 7, 10, or 13 months after treatment.

**Results:**

There was no significant difference in the primary end point between treatment arms (active, n = 46; control, n = 47; median difference, 14; 95% CI, −172.5 to 215.1; *P* = .80). Among secondary end points, nasal symptoms were worse in the intradermal treatment group, as measured based on daily (median difference, 35; 95% CI, 4.0-67.5; *P* = .03) and visual analog scale (median difference, 53; 95% CI, −11.6 to 125.2; *P* = .05) scores. In a per-protocol analysis intradermal immunotherapy was further associated with worse asthma symptoms and fewer symptom-free days. Intradermal immunotherapy increased serum *Phleum pratense*–specific IgE levels (*P* = .001) compared with those in the control arm. T cells cultured from biopsy specimens of subjects undergoing intradermal immunotherapy had higher expression of the T_H_2 surface marker CRTH2 (*P* = .04) and lower expression of the T_H_1 marker CXCR3 (*P* = .01), respectively. Late-phase responses remained inhibited 7 months after treatment (*P* = .03).

**Conclusion:**

Intradermal allergen immunotherapy suppressed skin late-phase responses but was not clinically effective and resulted in worsening of respiratory allergic symptoms.

Immunotherapy with grass pollen for seasonal allergic rhinitis is a longstanding and clinically effective treatment.^1^, ^2^ Conventional immunotherapy vaccines involve administration of high doses of allergen (typically 10- to 20-μg quantities of major allergens) by means of regular subcutaneous injection or as daily sublingual tablets, although both approaches have limitations. Subcutaneous immunotherapy is associated with a risk of systemic allergic reactions, and therefore injections require specialist supervision. Sublingual immunotherapy requires daily self-dosing for 3 years, and nonadherence is relatively commonplace.^3^

Intradermal allergen injection in sensitized subjects results in a localized wheal with erythema within 15 minutes (early-phase response), followed by diffuse indurated swelling that persists for 24 to 36 hours (late-phase response). The late-phase response is accompanied by infiltration of activated T_H_2 cells, eosinophils, and basophils, features that characterize chronic allergic inflammatory responses.^4^ We previously reported that repeated intradermal injections of grass pollen extract every 2 weeks lead to progressive and systemic attenuation of the macroscopic skin late-phase responses induced by these injections.^5^ After 6 intradermal injections, each containing the equivalent of 7 ng of the major allergen Phl p 5, late-phase responses were more than 90% suppressed, which is comparable with the degree of suppression achieved after conventional subcutaneous grass pollen immunotherapy containing more than 1000-fold greater cumulative allergen doses.

The concept of intradermal grass pollen allergen inoculation as a treatment for allergic rhinitis is not without precedent. In 1926, Phillips,^6^ a physician in Arizona, published a preliminary account of his experiences with intradermal grass pollen immunotherapy in 29 patients, which was extended to 322 patients by 1933,^7^ reporting that more than 90% obtained “satisfactory relief.” Here we report the findings of the first randomized placebo-controlled clinical trial of intradermal grass pollen injections for seasonal grass pollen allergy. The Pollen Low Dose Intradermal Therapy Evaluation (PollenLITE) study was conceived to test the hypothesis that skin late-phase response suppression after intradermal grass pollen administration is associated with clinical improvement in adults with seasonal allergic rhinitis.

## Methods

### Study design

**Fig 1:**
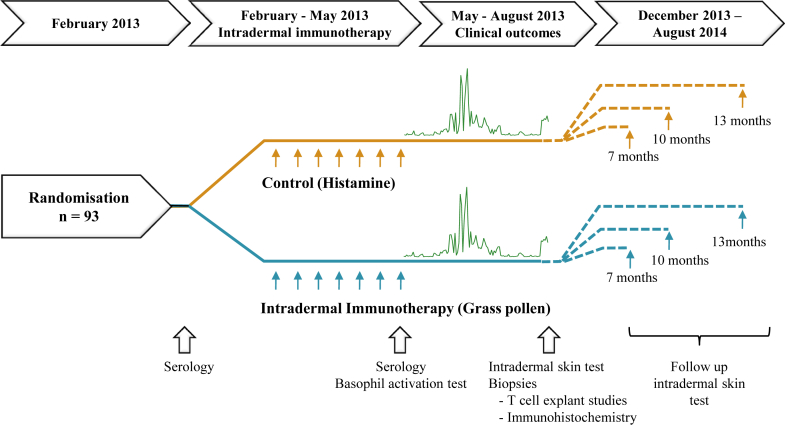
Study design.

PollenLITE was a single-center, randomized, placebo-controlled, double-blind phase 2 trial conducted at Guy's Hospital in London, investigating the efficacy and safety of 7 preseasonal intradermal injections of *Phleum pratense* (timothy grass) pollen extract versus a histamine control (Fig 1). The National Research Ethics Service Committee London-Harrow (12/LO/0941) and Medicines & Healthcare Products Regulatory Agency approved the study, with oversight by King's Health Partners Clinical Trial Office and an independent trial steering committee. The clinical trial protocol^8^ was finalized before randomization, and the statistical analysis plan was finalized before unblinding and data analysis. All participants provided written informed consent in accordance with the Declaration of Helsinki.

### Participant selection

Ninety-three participants were recruited by using advertisements in the press, online, and on public transport and a dedicated trial Web site. Eligible participants were aged 18 to 65 years with moderate-to-severe grass pollen–induced allergic rhinitis according to Allergic Rhinitis and Its Impact on Asthma (ARIA) classification,^9^ positive skin prick test responses (≥3 mm in diameter), and specific IgE levels (≥class 2) to *P pratense*. Exclusion criteria included seasonal grass pollen–induced asthma requiring regular albuterol or inhaled corticosteroids; symptomatic seasonal allergic rhinitis, asthma, or both caused by tree or weed pollen overlapping the grass season requiring regular treatment; and perennial rhinitis and previous life-threatening anaphylaxis. The full inclusion and exclusion criteria are described in the Methods section in this article's Online Repository at www.jacionline.org.

### Randomization

Participants were randomized 1:1 by the King's Clinical Trial Unit using block randomization with a 24-hour Web-based system, with stratification according to skin test response size to grass pollen and the presence of rhinitis symptoms outside the grass pollen season.

### Study procedures

Seven intradermal active or control histamine forearm injections were administered every 2 weeks before the 2013 grass pollen season (February 18 to May 24, 2013). Each active injection contained 10 biological units (BU) (33.3 SQ-U; 7 ng of the major allergen Phl p 5) of *P pratense* (Aquagen SQ Timothy; ALK-Abelló, Reading, United Kingdom) in a 20-μL volume. This regimen was chosen based on our previous study showing that 6 injections at the same dose and interval led to 90% suppression of the late-phase response in the skin. Histamine control was administered at 100 μg/mL for the first 2 injections, reduced to 30 μg/mL for the second 2 injections, and then reduced 10 μg/mL for the final injections to help preserve blinding. Details of active and placebo manufacture are supplied in the Methods section in this article's Online Repository. Antihistamines were avoided 5 days before intradermal injections, so that a wheal in response to the injection could be confirmed. All participants were observed for systemic reactions after the first injection for 1 hour and for 30 minutes after subsequent injections. Participants completed diary cards during the 2013 grass pollen season, recording symptoms and rescue medication use.

### Study outcomes

The primary outcome was a combined symptom and medication score during the grass pollen season (May 13 to August 31, 2013; 111 days), as recommended by World Allergy Organization (WAO) guidelines for allergic rhinitis immunotherapy trials (see the Methods section in this article's Online Repository for details of symptom and medication scoring).^10^

Predefined secondary clinical end points were overall symptom scores; individual nose, mouth, eye, and lung symptom scores; overall medication scores; combined symptom and medication scores during the peak season; visual analog scale (VAS) scores for nose and eye symptoms (every 2 weeks); mini-Rhinoconjunctivitis Quality of Life Questionnaire (mini-RQLQ) and health-related quality of life (EQ-5D-5L) scores (4 time points); a global evaluation of symptoms (at the end of the season); number of symptom and medication-free days; and number of days prednisone was used. Adverse events were recorded for all patients who received at least 1 dose of study drug (see the Methods section in this article's Online Repository). To verify blinding, participants guessed whether they had received the active or control intervention after the 2013 pollen season.

In September 2013 (ie, 4 months after completion of intradermal treatment injections), cutaneous early-phase (15 minutes) and late-phase (24 hours) responses were measured after intradermal injections of grass pollen (identical to treatment dose) and diluent (ALK-Abelló). Twenty participants per treatment arm were also randomized to undergo 3-mm punch biopsies from these sites after 24 hours. Biopsy specimens were all analyzed by means of immunohistochemistry for numbers of eosinophils, neutrophils, CD3^+^ T cells, and CD4^+^ T cells. In half of participants who underwent biopsy, the biopsy specimens were divided into 2 fragments, with the second fragment used for T-cell expansion, flow cytometric evaluation of T_H_1/T_H_2 markers, and microarray analysis. Blood specimens were collected for *P pratense*–specific IgE and IgG levels and basophil activation studies. Subjects were also randomized for repeat late-phase response measurements at either 7, 10, or 13 months after treatment completion. Further methodological information is provided in the Methods section in this article's Online Repository.

### Statistical analysis

Details of the power calculation are provided in the Methods section in this article's Online Repository. All analyses were predefined in a detailed statistical analysis plan and overseen by a data monitoring committee. Primary outcome analysis, performed on an intention-to-treat basis, included all participants who were randomized without imputation for missing data. Differences between the groups in the area under the curve (AUC) of combined symptom and medication scores, the primary outcome, were assessed by using a stratified Mann-Whitney *U* test (van Elteren test) adjusted for baseline stratification factors. The stratified Hodges-Lehmann estimation was used to calculate median differences with CIs. Similar analyses were conducted for total and organ symptom scores, medication scores, and VAS scores. Mini-RQLQ and EQ-5D-5L scores were evaluated by using linear mixed models with 95% CIs. Sensitivity analyses were performed with missing data imputed by using mean scores on the day concerned and in the relevant trial arm for primary and secondary outcomes in the intention-to-treat population. Analyses were also performed in the predefined per-protocol population. All mechanistic analyses were performed with the Mann-Whitney *U* test, except serology and immunohistochemistry, which were analyzed by means of analysis of covariance. The Wilcoxon signed-rank test was used to compare pretreatment versus posttreatment serology and diluent control versus allergen challenge immunohistochemistry results.

The principal software package was SAS/STAT (SAS Institute, Cary, NC), with verification of results from Syntax for selected analyses analyzed in Stata (StataCorp, College Station, Tex). This trial was registered with Current Controlled Trials (no. ISRCTN 78413121).

## Results

### Study participants

**Fig 2:**
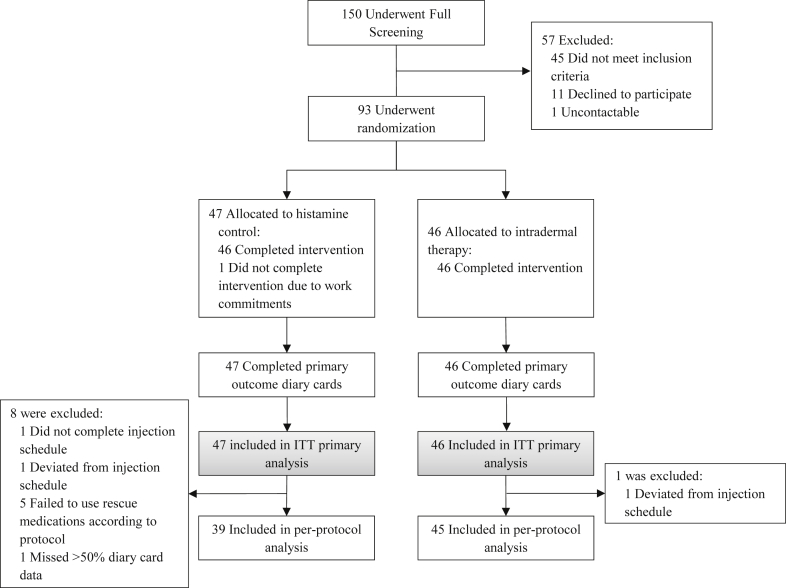
CONSORT diagram. All randomized participants were included in the intention-to-treat *(ITT)* analysis. Only participants who adequately adhered to treatment and rescue medications were included in the per-protocol analysis.

A total of 93 participants were randomized. All could be evaluated for the primary outcome in the intention-to-treat analysis (Fig 2). Baseline characteristics were well balanced between groups (Table I). All 46 participants receiving intradermal allergen immunotherapy completed the treatment course; one delayed an injection by 1 day because of a scheduling conflict. One of 47 participants assigned to control injections withdrew after the second injection because of work commitments, and another delayed an injection by 4 days because of an upper respiratory tract infection. Missing diary data for the primary end point were few, with 94% of participants supplying more than 90% of daily data. One patient completed less than the predetermined per-protocol 50% threshold of daily data and was excluded from the per-protocol population. Five participants, all in the control arm, significantly deviated from protocol-specified use of rescue medications. After the pollen season, participants were unable to identify whether they had received active allergen or histamine control treatment (see Table E1 in this article's Online Repository at www.jacionline.org).

**Table I:** Baseline characteristics of study participants

Characteristic	Control subjects (n = 47)	Subjects receiving intradermal immunotherapy (n = 46)
Age (y), mean (SD)	35 (10.8)	32 (9.9)
Female sex, no. (%)	12 (26)	19 (41)
Race, no. (%)		
White	37 (79)	37 (80)
Mixed	2 (4)	3 (7)
Asian	3 (6)	4 (9)
Black	3 (6)	0 (0)
Other	2 (4)	2 (4)
Allergy symptoms outside grass pollen season, no. (%)	18 (38)	16 (35)
Total IgE (kU/L), median (IQR)	121 (64-255)	160 (80-263)
*P pratense*–specific IgE (kU_A_/L), median (IQR)	27 (10-54)	22 (9-49)
*P pratense*–specific SPT wheal diameter (mm), mean (SD)	12 (4.2)	11 (5.0)
Positive SPT response, no. (%)		
Timothy grass	47 (100)	46 (100)
Mixed grass	47 (100)	46 (100)
Silver birch	19 (40)	24 (52)
Mugwort	11 (23)	9 (20)
House dust mite	28 (60)	24 (52)
Cat	24 (51)	18 (39)
Dog	41 (87)	36 (78)
Horse	4 (9)	6 (13)
*Aspergillus* species	1 (2)	2 (4)
*Alternaria* species	6 (13)	7 (15)
*Cladosporium* species	2 (4)	2 (4)
Seasonal asthma controlled with albuterol	17 (36)	15 (33)

*SPT*, Skin prick test.

### Primary outcome

**Fig 3:**
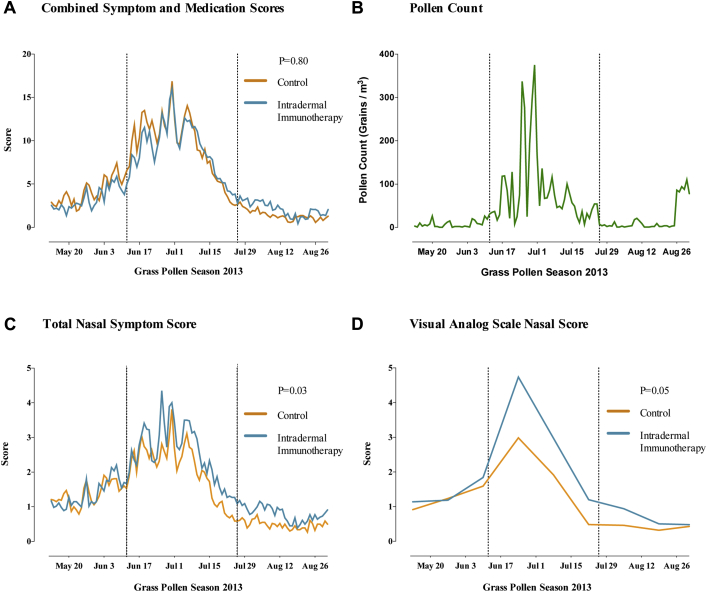
Primary outcome and nasal symptoms. **A,** Mean daily combined symptom and medication scores in the primary intention-to-treat analysis. *Broken vertical lines* indicate the beginning and end of the peak pollen season (June 12 to July 26, 2013). **B,** Daily grass pollen counts in central London during the 2013 grass pollen season. **C,** Mean daily nasal symptom scores (sum of scores for sneezing, blockage, and running). **D,** Mean nasal symptoms measured by using a VAS (total of blockage, running, itching, and sneezing). AUC values for each participant were compared according to treatment arm. *P* values are based on the Mann-Whitney *U* test.

There was a clear temporal relationship between the combined symptom and medication scores and daily pollen counts (Fig 3, *A*), which peaked at above-average levels. Intradermal immunotherapy did not significantly affect the primary end point (ie, the combined symptom and medication score over the entire grass pollen season [111 days]; difference in median AUC, 14; 95% CI, −172.5 to 215.1; *P* = .80; Fig 3, *B*; Table II).

**Table II:** Effect of intradermal immunotherapy on primary and secondary outcomes (intention-to-treat analysis)

Clinical outcome	Control subjects (n = 47), median (IQR)	Subjects receiving intradermal immunotherapy (n = 46), median (IQR)	Difference (95% CI)	*P* value
Primary outcome				
CSMS during entire season	487 (365-717)	502 (333-841)	14 (−172.5 to 215.1)	.80
Secondary outcomes				
Symptom score during entire season	264 (156-398)	335 (183-503)	59 (−1.3 to 110.9)	.24
Medication score during entire season	263 (129-482)	242 (116-405)	−19 (−153.0 to 100.2)	.44
CSMS during peak season	365 (278-508)	356 (232-521)	−8 (−75.8 to 66.3)	.90
Nasal symptom score during entire season	121 (81-200)	174 (120-207)	35 (4.0 to 67.5)	.03
Mouth symptom score during entire season	14 (5-45)	34 (8-90)	10 (3.8 to 24)	.05
Eye symptom score during entire season	78 (52-180)	79 (41-153)	−7 (−18.5 to 2.9)	.54
Lung symptom score during entire season	12 (0-34)	17 (3-32)	4 (−1 to 15)	.17
Nasal allergic symptoms measured by VAS	122 (54-184)	156 (104-275)	53 (−11.6 to 125.2)	.05
Eye allergic symptoms measured by VAS	144 (41-176)	84 (32-197)	−3 (−46.0 to 35.8)	.40
Global evaluation of symptom scores	3 (1-4)	3 (2-4)	0 (0 to 1)	.48
Symptom-free days	41 (23-61)	35 (19-53)	−6 (−17 to 3)	.15
No. of days prednisone used during entire season	0 (0-0)	0 (0-0)	0 (0 to 0)	.36
Medication-free days	76 (65-94)	81 (65-93)	4 (−11 to 21)	.22
Mini-RQLQ	18 (10-25)	16 (13-23)	−0.3 (−4.2 to 3.7)	.89
EQ-5D-5L	88 (81-94)	87 (83-94)	9 (−24.8 to 43.6)	.59

Median difference between groups was calculated by using stratified Hodges-Lehmann estimation. *P* values were based on the stratified Mann-Whitney *U* (Van Elteren) test adjusted for stratification factors. *P* values for mini-RQLQ and EQ-5D-5L scores were based on linear mixed model adjusted for stratification factor. The entire grass pollen season was from May 13-August 31, 2013; the peak season was from June 12-July 26, 2013.

*CSMS*, Combined symptom and medication score; *EQ-5D-5L*, EuroQoL instrument.

### Secondary outcomes

No significant group differences were seen in secondary end points of overall symptom scores (*P* = .24) and rescue medication use (*P* = .44) during the whole season and combined symptom and medication scores during the peak season (June 12 to July 26, 2013; *P* = .90; Table II).

Among other secondary end points, allergic rhinitis symptoms measured based on daily nasal symptom scores were 44% higher in the intradermal allergen immunotherapy group, with a difference in median AUC of 35 (95% CI, 4.0-67.5; *P* = .03; Fig 3, *C*). Rhinitis symptoms measured by using a VAS were 28% higher in the intradermal allergen immunotherapy group, with a difference in median AUC of 53 (95% CI, −11.6 to 125.2; *P* = .05; Fig 3, *D*). No significant differences were seen between groups in daily eye or lung symptoms (Table II), although mouth symptoms tended to be more frequent in the intradermal allergen group (median difference in AUC, 10.0; 95% CI, 3.8-24; *P* = .05). No significant group differences were observed in eye symptoms measured by using VAS scores, mini-RQLQ scores, EQ-5D-5L scores, global evaluation of symptoms scores, number of symptom or medication-free days, or number of days prednisone was taken.

In the per-protocol analysis (Table III) the individual nasal (*P* = .02) and mouth (*P* = .02) daily symptom scores were significantly higher in the active group, whereas lung daily symptom scores (*P* = .05) and overall symptom scores (*P* = .09) tended toward significance. Active group participants also had significantly worse nasal symptoms measured by using VASs (*P* = .008) and recorded fewer symptom-free days than subjects in the control group (*P* = .04). In the intention-to-treat analysis, when missing data were imputed (see Table E2 in this article's Online Repository at www.jacionline.org), nasal daily symptom scores (*P* = .03) and VAS nasal symptom scores were statistically significant (*P* = .02), and mouth symptoms tended to be higher (*P* = .05).

**Table III:** Effect of intradermal immunotherapy on primary and secondary outcomes (per-protocol sensitivity analysis)

Clinical outcome	Control subjects (n = 39), median (IQR)	Subjects receiving intradermal immunotherapy (n = 45), median (IQR)	Difference (95% CI)	*P* value
Primary outcome				
CSMS during entire season	453 (279-685)	517 (344-841)	82 (−121.8 to 280.1)	.23
Secondary outcomes				
Symptom score during entire season	241 (150-398)	340 (189-503)	76 (25.9 to 133.5)	.09
Medication score during entire season	254 (113-358)	255 (119-405)	21 (−125.0 to 157.0)	.83
CSMS during peak season	342 (242-476)	363 (242-546)	18 (−73.2 to 127.5)	.51
Nasal symptom score during entire season	119 (80-205)	173 (123-207)	40 (13.3 to 71.5)	.02
Mouth symptom score during entire season	14 (4-43)	38 (8-90)	14 (4.9 to 32.0)	.02
Eye symptom score during entire season	72 (48-145)	80 (41-153)	0 (−16.0 to 17.6)	.85
Lung symptom score during entire season	11 (0-21)	17 (3-32)	9 (1.0 to 17.0)	.05
Nasal allergic symptoms measured by VAS	118 (50-154)	162 (105-275)	68 (8.3 to 134.6)	.008
Eye allergic symptoms measured by VAS	114 (42-159)	90 (32-197)	1 (−52.8 to 62.0)	.49
Global evaluation of symptom scores	3 (1-3)	3 (2-4)	1 (0.0 to 1.0)	.25
Symptom-free days	44 (25-67)	34 (19-47)	−12 (−22.0 to −2.0)	.04
No. of days prednisone used during entire season	0 (0-0)	0 (0-0)	0 (0 to 0)	.33
Medication-free days	78 (66-98)	80 (65-92)	−1 (−20.0 to 17.0)	.87
Mini-RQLQ	17 (10-22)	16 (13-23)	−2.0 (−5.89 to 1.88)	.31
EQ-5D-5L	88 (84-94)	88 (83-94)	3 (−28.4 to 35.2)	.83

Data for primary outcome and all symptom scores represent AUC values. Median difference between groups was calculated by using stratified Hodges-Lehmann estimation. *P* values are based on the stratified Mann-Whitney *U* (Van Elteren) test adjusted for stratification factors. *P* values for mini-RQLQ and EQ-5D-5L scores were based on a linear mixed model adjusted for stratification factors. The entire grass pollen season was from May 13-August 31, 2013; the peak season was from June 12-July 26, 2013.

*CSMS*, Combined symptom and medication score; *EQ-5D-5L*, EuroQoL instrument.

Because allergic rhinitis nasal symptoms were unexpectedly worse in intradermal immunotherapy participants, we performed *post hoc* analyses comparing daily data for each individual allergic symptom between groups (Table IV). In the active group scores for sneezing (*P* = .01), cough (*P* = .02), chest tightness (*P* = .08), and mouth itching (*P* = .06) were higher, whereas eye swelling scores were lower (*P* = .03). Individual nasal symptoms measured by using VAS scores also revealed higher scores after intradermal immunotherapy for rhinorrhea (*P* = .006), sneezing (*P* = .006), and nasal itching (*P* = .003, Table IV).

**Table IV:** Effect of intradermal immunotherapy on daily and VAS organ symptom scores (intention-to-treat and *post hoc* analysis)

Individual symptom	Control subjects (n = 47), median (IQR)	Intradermal immunotherapy (n = 46), median (IQR)	Difference (95% CI)	*P* value
Daily organ symptom scores				
Nose				
Sneezing	55 (35.0-71.0)	76 (43.3-103.0)	21 (7.0 to 34.0)	.01
Blockage	36 (12.5-61.0)	41 (14.0-74.5)	6 (−2.5 to 13.5)	.33
Running	46 (22.5-65.4)	51 (30.0-81.5)	10 (−3.0 to 22.8)	.17
Mouth				
Itching	8 (1.0-25.0)	19 (4.0-52.3)	4 (1.8 to 6.8)	.06
Drying	3 (0.0-15.0)	7 (0.0-40.0)	3 (0.0 to 9.6)	.18
Eyes				
Itching	44 (26.0-72.5)	48 (21.0-68.0)	−1 (−5.0 to 2.0)	.99
Redness/sore	14 (7.0-45.0)	17 (4.0-42.0)	−1 (−6.0 to 3.0)	.55
Streaming	14 (2.0-24.0)	11 (2.0-19.0)	0 (−4.0 to 3.0)	.69
Swelling	5 (0.0-14.0)	2 (0.0-9.0)	−2 (−4.0 to 0.0)	.03
Lungs				
Breathlessness	0 (0.0-8.1)	0 (0.0-4.0)	0 (0.0 to 2.0)	.27
Cough	1 (0.0-12.1)	8 (1.0-23.3)	2 (0.0 to 6.0)	.02
Wheezing	0 (0.0-8.0)	3 (0.0-7.0)	0 (0.0 to 2.0)	.25
Tightness	0 (0.0-4.0)	2 (0.0-4.0)	0 (0.0 to 2.0)	.08
VAS organ symptom scores				
Nose				
Blockage	118 (39.1-178.8)	152 (71.4-238.7)	39 (1.6 to 82.8)	.12
Running	117 (62.0-162.7)	169 (96.0-265.6)	58 (−8.2 to 124.5)	.006
Itching	81 (41.9-141.6)	138 (93.2-281.7)	64 (−16.3 to 165.4)	.003
Sneezing	125 (46.1-182.4)	187 (133.1-295.3)	77 (−1.6 to 150.9)	.006
Eyes				
Itching	135 (41.9-217.8)	120 (53.7-248.3)	4 (−35.3 to 46.1)	.97
Watering	71 (33.6-119.4)	69 (21.0-129.5)	1 (−40.5 to 55.5)	.79

Data shown represent AUC values. Median difference between groups was calculated by using stratified Hodges-Lehmann estimation. *P* values were based on the stratified Mann-Whitney *U* (Van Elteren) test adjusted for baseline stratification factors.

The frequency of adverse events was similar between groups. The frequency of treatment-related adverse events was low: 3 (6.5%) and 6 (13%) participants in the intradermal immunotherapy and control groups, respectively, experienced mild systematic reactions manifested as generalized pruritus only, except for 1 participant receiving intradermal allergen who had erythema tracking from the injection site in a lymphatic distribution (IgE-mediated lymphangitis) 20 minutes after each injection. There were 3 serious adverse events all unrelated to treatment: 1 (2.2%) in the active group and 2 (4.3%) in the control group (see Table E3 in this article's Online Repository at www.jacionline.org).

### Immunologic findings

**Fig 4:**
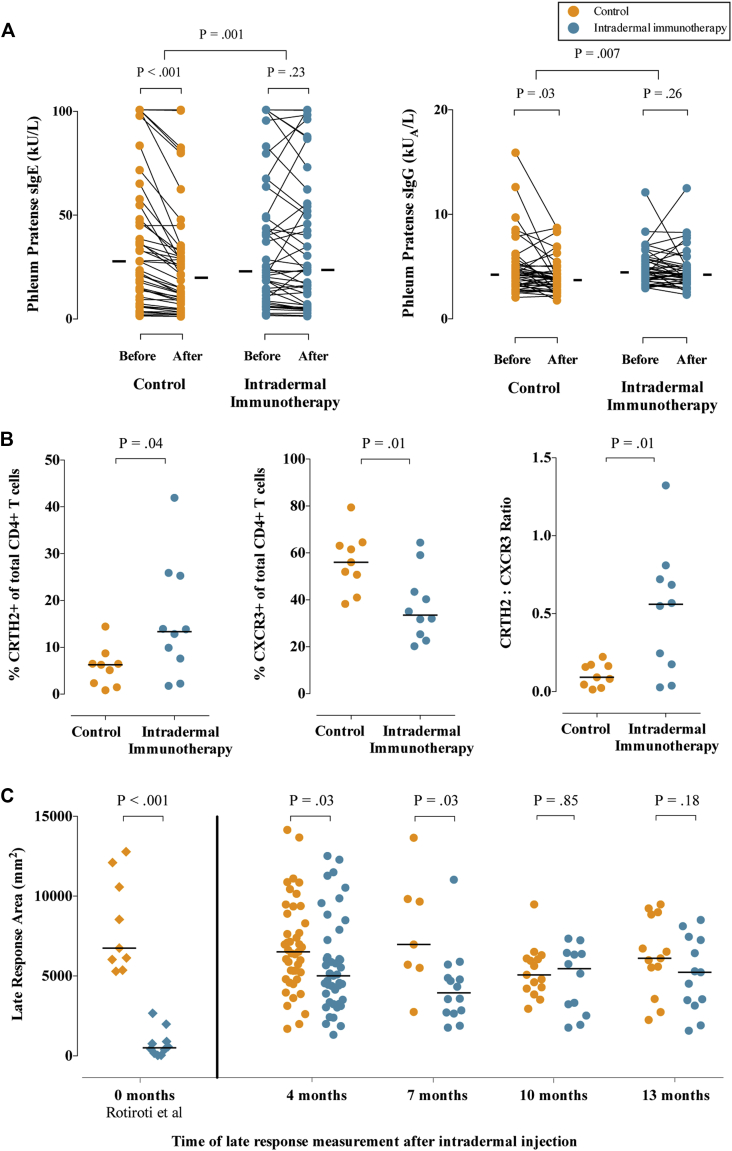
Immunologic outcomes. **A,** Levels of *P pratense*–specific IgE and IgG before and after completion of 7 intradermal allergen or histamine control injections. **B,** Expression of CRTH2 (T_H_2 marker) and CXCR3 (T_H_1 marker) on CD4^+^ cells expanded from skin biopsy specimens (24 hours after skin challenge). **C,** Areas of cutaneous late-phase responses (24 hours after intradermal skin challenge) 4 months and either 7, 10, or 13 months after treatment (September 2013). Late-phase response suppression was shown in our previous study (Rotiroti et al^5^) immediately after 6 biweekly intradermal injections. *Solid bars* represent median values. *P* values for pretreatment and posttreatment serology comparisons are based on the Wilcoxon signed-rank test, and those for between-group IgE and IgG levels are based on analysis of covariance. *P* values in Fig 4, *B* and *C*, are based on the Mann-Whitney *U* test.

**Fig E1:**
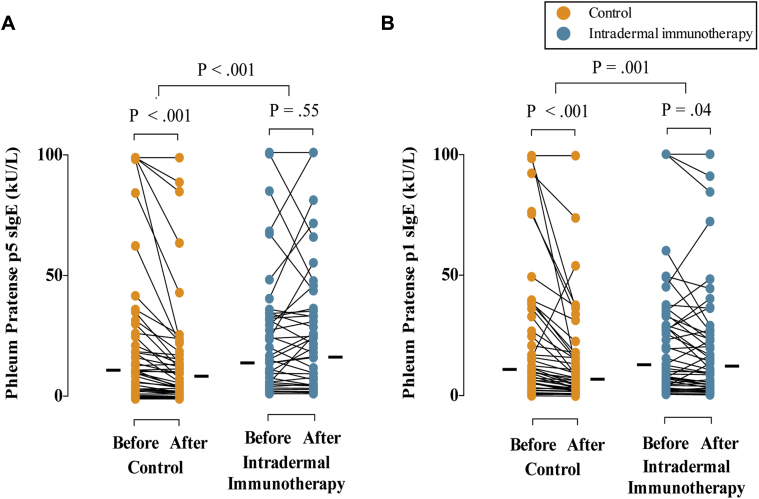
Effects of intradermal immunotherapy on Phl p 5– **(A)** and Phl p 1– **(B)** specific IgE. Levels of IgE specific for the major allergens Phl p 5 and Phl p 1 before and after completion of 7 intradermal allergen or histamine control injections are shown. *P* values for pretreatment and posttreatment comparisons were based on the Wilcoxon signed-rank test. *P* values for between-group comparisons were based on analysis of covariance.

Serologic assessments before (October 2012) and after (May 2013) treatment showed a typical seasonal decrease in allergen-specific IgE levels in the control group (*P* < .001), which was significantly less in the intradermal allergen immunotherapy group (*P* = .001), indicating a treatment-induced relative increase in allergen-specific IgE levels (Fig 4, *A*). A treatment effect was also seen on *P pratense*–specific IgG (*P* = .03; Fig 4, *B*) and IgE titers to the major grass allergens Phl p 5 and Phl p 1 (see Fig E1 in this article's Online Repository at www.jacionline.org), although no effect was seen on IgG_4_ responses (data not shown).

**Fig E2:**
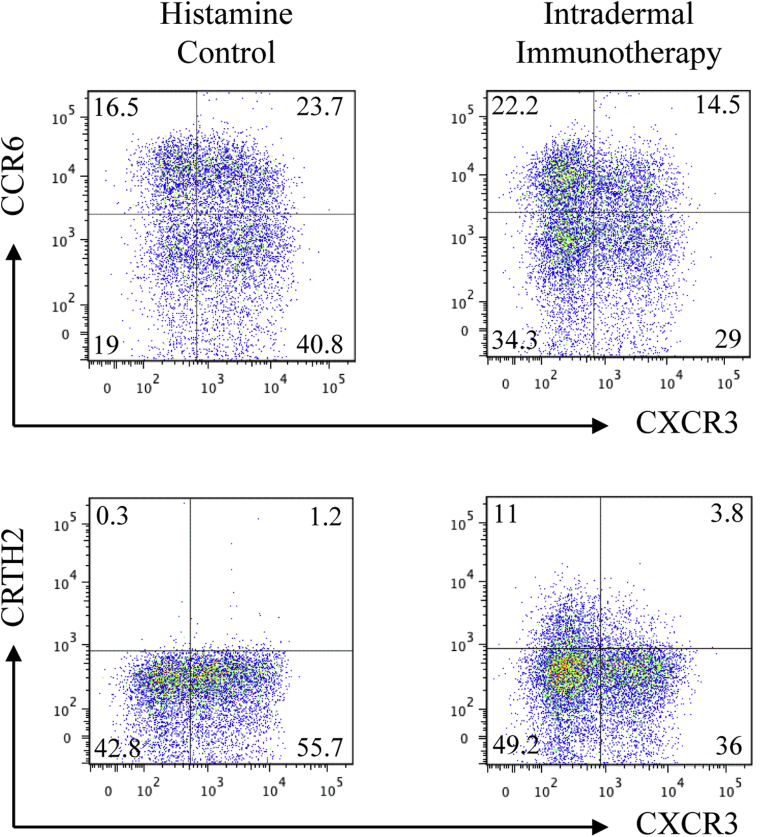
Flow cytometric analysis of CD4^+^ T cells from skin biopsy explants. Representative flow cytometric plots illustrating surface staining for CCR6, CXCR3, and CRTH2 gated on skin biopsy-derived CD4^+^ T cells in a participant who received histamine control *(left)* and a participant who received grass pollen intradermal injections *(right)* are shown.

**Fig E3:**
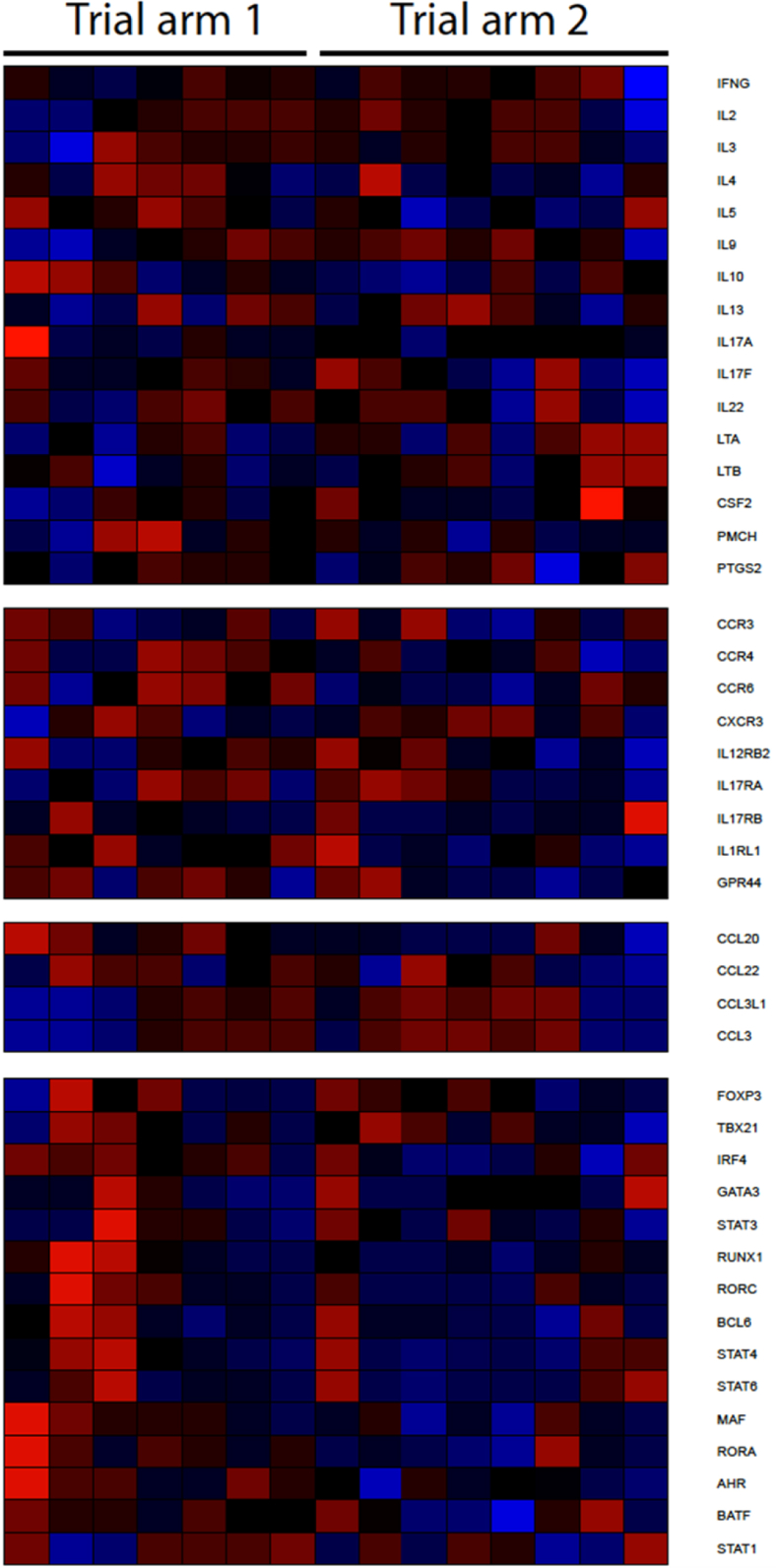
Heat map showing expression of selected genes associated with T_H_1/T_H_2 phenotypes and allergic inflammatory responses.

**Fig E4:**
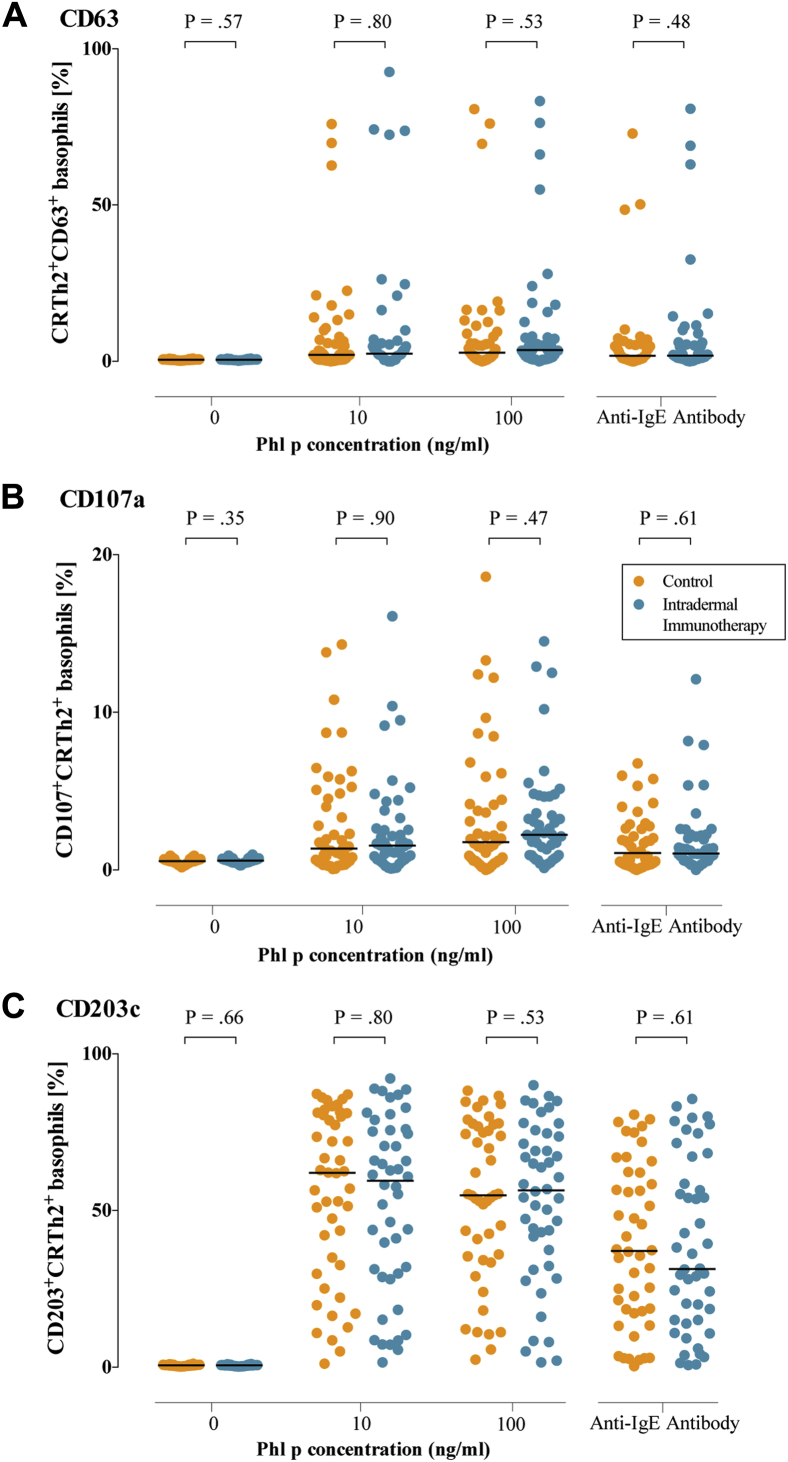
Basophil activation tests. Percentages of basophils staining positive for the activation markers CD63 **(A)**, CD107a **(B)**, and CD203c **(C)** are shown. Whole blood was stimulated under the conditions shown. *P* values are based on the Mann-Whitney *U* test.

**Fig E5:**
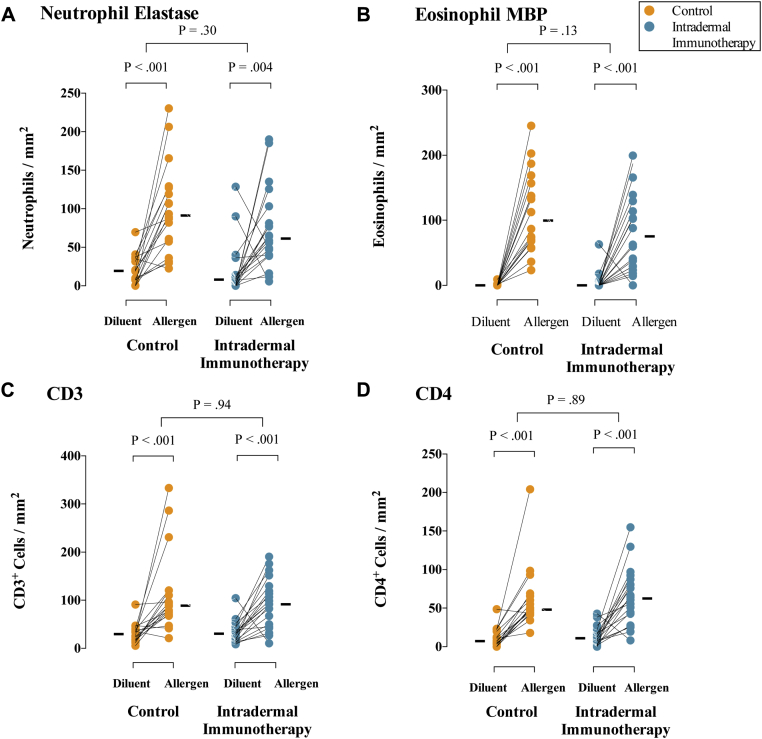
Immunohistochemistry analysis of skin biopsy specimens. Comparison of allergen-induced inflammatory cell numbers in skin biopsy specimens from intradermal immunotherapy and control arm participants. Data shown indicate numbers of neutrophils **(A)**, eosinophils **(B)**, CD3^+^ cells **(C)**, and CD4^+^ cells **(D)** in skin biopsy specimens taken after diluent and *P Pratense* intradermal skin challenges in September 2013. Cells were stained by using the APAAP method. *Solid bars* represent median values. *P* values comparing diluent- and allergen-challenged biopsy specimens are based on the Wilcoxon signed-rank test. *P* values for between-group comparisons are based on analysis of covariance.

CD4^+^ T cells expanded from 19 of 20 skin biopsy specimens collected after intradermal grass pollen challenge after the 2013 grass pollen season showed higher expression of the T_H_2 marker chemoattractant receptor-homologous molecule expressed on T_H_2 lymphocytes (CRTH2) in the active group (median, 13.4%; interquartile range [IQR], 6.3% to 25.4%) compared with the control group (median, 6.3%; IQR, 1.9% to 7.6%; *P* = .04), whereas expression of the T_H_1 cell marker CXCR3 was lower (median, 33.5% [IQR, 24.7% to 47.3%] vs 56% [IQR, 45.8% to 63.8%]; *P* = .01; Fig 4, *B*, and see Fig E2 in this article's Online Repository at www.jacionline.org). No differences were seen in expression of the T_H_17 marker CCR6 (data not shown). Insufficient T cells could be expanded from diluent-challenged skin biopsy specimens for analysis. Microarray transcriptional profiling performed on cultured T cells from 15 allergen-challenged skin biopsy specimens showed only 14 genes that were significantly overexpressed in the active group (defined as >1.5-fold higher expression than the control group and *P* < .05 by using a 3-way ANOVA model), including IL-5, but no other T_H_2- or T_H_1-related genes (see Table E4 in this article's Online Repository at www.jacionline.org; microarray Gene Expression Omnibus Accession no. GSE72324; see Fig E3 in this article's Online Repository at www.jacionline.org for heat map of cytokines and relevant transcription factors). Gene ontology analysis did not highlight a broader effect on T_H_2 or inflammation-related genes. No significant treatment effect was seen on surface expression of peripheral blood basophil activation markers (see Fig E4 in this article's Online Repository at www.jacionline.org) or on numbers of eosinophils, neutrophils, CD3^+^ T cells, and CD4^+^ T cells after immunohistochemical staining of diluent- and allergen-challenged skin biopsy specimens (see Fig E5 in this article's Online Repository at www.jacionline.org).

### Skin challenge results

Early-phase (15 minutes) and late-phase (24 hour) skin responses could be measured in 86 participants 4 months after the final intradermal allergen injection (September 2013) and then repeated at either 7, 10, or 13 months. The size of the late-phase responses in the control group was consistent with that reported in our previous study under the same conditions (shown for comparison in Fig 4, *C*).^5^ In the present trial the late-phase response was still suppressed 4 and 7 months after completing intradermal allergen treatment (*P* = .03 for both time points) but not at 10 or 13 months. In comparison with the historical data, however, suppression at these times was less than that which we observed immediately after completing 6 injections (Fig 4, *C*), suggesting that the suppressive effect on late-phase responses was wearing off within 4 months.

## Discussion

In this phase 2, randomized, double-blind, placebo-controlled trial in adults with moderate-to-severe allergic rhinitis, preseasonal treatment with intradermal grass pollen injections did not affect the primary end point (combined symptom and medication scores during the 2013 grass pollen season). These findings repudiate our hypothesis that suppression of cutaneous late-phase responses after repeated intradermal low-dose grass pollen injections^5^ would be associated with clinical improvement of allergic rhinitis. Intradermal allergen immunotherapy was associated with 44% worse allergic rhinitis nasal symptoms, as measured by daily symptom scores, and 28% worse symptoms, as measured by VAS scores, although the trial was neither designed nor powered to detect deterioration of symptoms. These findings were consistent when missing data were imputed. In the per-protocol population, in addition to worsening of nasal symptoms measured both daily and by VAS scores, we found worsening of lung and mouth symptoms and significantly fewer symptom-free days.

No serious adverse events attributable to grass pollen intradermal allergen immunotherapy occurred. Ninety-two of the 93 participants completed the full injection course; 1 withdrew for unrelated reasons. Five participants deviated significantly from the protocol in use of rescue medications, mainly using excessive antihistamines, topical nasal steroids, or eye drops. Two of these participants also used prednisone without study physician guidance. We are unable to account for why these 5 participants were all in the control arm, although their exclusion from the per-protocol population did not affect the conclusions of the study.

The strengths of this first randomized controlled trial of low-dose intradermal immunotherapy include recruitment of participants with moderate-to-severe symptoms in accordance with ARIA classification; use of the primary outcome of combined symptom and medication scores during the grass pollen season in accordance with WAO guidance for allergic rhinitis trials; a low level of missing daily diary card data; and successful blinding of the active treatment. This was achieved through daily data entry by participants, text reminders, and regular data collection throughout the season.

**Fig E6:**
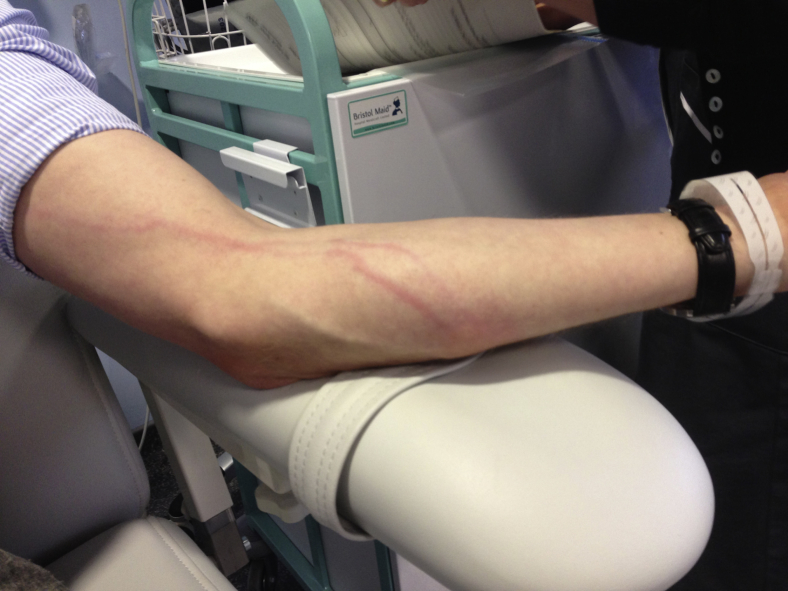
Lymphangitis in a participant who received active intradermal immunotherapy. The photograph was taken 40 minutes after intradermal injection.

The rationale for a trial of intradermal immunotherapy was based on our previous study^5^ showing that this regimen systemically abrogated allergen-induced skin late responses and also previous clinical studies suggesting that epicutaneous^11^, ^12^, ^13^ and intralymphatic^14^, ^15^ immunotherapy might be clinically effective. We hypothesized that intradermal injection of allergen might promote tolerogenic pathways through rapid uptake to regional lymph nodes or possibly by dermal dendritic cell (DC) populations, which are relatively abundant compared with subcutaneous tissue.^16^ Indeed, one of our active group participants reproducibly demonstrated lymphangitis (see Fig E6 in this article's Online Repository at www.jacionline.org) within 30 minutes of each injection, which is suggestive of rapid lymphatic uptake of allergen. We selected an allergen dose equivalent to 7 ng of the major timothy grass pollen allergen Phl p 5 for several reasons.

First, we previously reported in a proof-of-concept study conducted in a similar population that 6 biweekly injections at the same dose led to almost complete attenuation of the cutaneous late-phase response induced by these injections. This is comparable with the effect on cutaneous late-phase responses seen after high-dose subcutaneous immunotherapy^17^ and exceeds that after treatment with sublingual grass pollen vaccines.^18^

Second, the average late-phase response induced by this dose was approximately 10 cm in diameter, which we considered to be at the limits of tolerability for patients. Although precise intradermal grass dosages used in the uncontrolled historic studies of Phillips are unknown,^6^, ^7^ his aim during treatment was to induce “a local reaction about the size of the patient's palm, which should begin to subside within twenty four hours.”

Our study has possible limitations. First, grass pollen doses were not increased during the treatment course. This treatment protocol was chosen because of our previous observation that repeating the same dose was sufficient to achieve almost complete suppression of the late-phase response.

Second, injections were not continued throughout the grass pollen season, although previous randomized controlled trials of subcutaneous grass pollen immunotherapy have demonstrated efficacy for preseasonal regimens.^19^

Late-phase skin responses were first measured at the end of the 2013 grass pollen season because performing such measurements before or during collection of clinical outcome data would have risked unblinding the trial. Late-phase responses still appeared partially suppressed at this and the subsequent 7-month time points. Nonetheless, this difference was less than we observed immediately after completion of 6 intradermal injections in the proof-of-concept study, suggesting that suppression is transient and mostly reversed within 4 months. Therefore this effect might be similar to that seen with transient desensitization during food oral immunotherapy. The late cutaneous response is considered to be at least partially T cell dependent and has been extensively used as an experimental model for exploring mechanisms of allergic disease.^4^, ^20^ Our data suggest that either the late-phase skin response is not relevant for disease expression or, more likely in our view, that suppression of the late-phase response might be necessary but not sufficient for clinical improvement after allergen-specific immunotherapy.

The decrease in *P pratense*–, Phl p 1–, and Phl p 5–specific IgE levels in the placebo group between the baseline (October 2012) and follow-up measurement after 7 injections (May 2013) was consistent with natural seasonal variation, as described in previous studies; levels of pollen-specific IgE increase during the grass pollen season and then gradually decrease over the following winter months.^21^, ^22^ Similar changes also occur in pollen-specific IgG antibodies.^22^ Intradermal immunotherapy arrested the anticipated winter decrease, which was seen in the placebo group. Therefore, taking into account the seasonal changes, intradermal allergen immunotherapy stimulated IgE production. In keeping with this and the exacerbation of nasal symptoms (and other clinical parameters in the per-protocol population), T cells cultured from skin punch biopsy explants in the intradermal immunotherapy group expressed higher levels of the T_H_2 marker CRTH2 and lower surface expression of the T_H_1 marker CXCR3 than biopsy specimens from placebo-treated subjects. Exploratory microarray analysis of these T cells was performed in a subgroup only because of limited cell numbers. Although IL-5 was one of only 14 genes overexpressed according to prespecified criteria, gene ontology analysis did not highlight an effect on other T_H_2- or inflammation-related genes. Also, *post hoc* analysis with less stringent criteria did not highlight additional T_H_2- or T_H_1-related genes. Therefore, although the clinical and other immunologic findings indicate a priming effect, we interpret the IL-5 microarray data in isolation with caution.

An intradermal priming effect could be consistent with observational human studies linking cutaneous exposure to peanut protein in children with atopic dermatitis with development of peanut allergy, an effect more apparent in those with impaired skin barrier function, which might promote dermal allergen exposure.^23^, ^24^ Our findings also raise the possibility that intracutaneous exposure to aeroallergens, for example in patients with atopic dermatitis with disrupted skin barrier function, might have potential to promote or exacerbate respiratory allergic disease. Such a link has been hypothesized as the basis of so-called “atopic march” from atopic dermatitis to later development of respiratory allergies.^25^

Previous attempts to develop novel immunotherapy approaches based on epicutaneous allergen application have shown some initial promise. Early-phase clinical trials have provided evidence that allergen patches be effective for treatment of grass pollen allergy,^13^ and similar patches are also under investigation for peanut allergy.^11^, ^12^ A potentially important immunologic difference between epicutaneous and intradermal allergen immunotherapy is in the types of antigen-presenting cells, particularly DC populations, likely to be encountered by allergen.^16^ In the epidermis Langerhans cells predominate, although atopy patch tests also induce infiltration by inflammatory dendritic epidermal cells,^26^ whereas in the dermis 3 major DC subtypes have been identified.^27^ Recent attention has focused on methods that enhance keratinocyte activation and skin penetration by epicutaneous allergen, such as skin stripping^28^ or use of microneedles.^29^ Skin barrier disruption appears to promote dermal allergen exposure,^30^ and in some animal models epicutaneous immunotherapy on stripped skin has appeared to potentiate pre-existing systemic TH2 responses.^31^ More recently, dermal DCs, but not Langerhans cells, were found to elicit murine T_H_2 responses in response to epicutaneous antigen.^32^

In conclusion, this is the first randomized controlled trial to directly evaluate the efficacy of intradermal grass pollen immunotherapy, and the results suggest that this approach is not clinically effective, despite local suppression of skin late-phase responses. Moreover, the data suggest that this resulted in immunologic priming and worsening of allergic rhinitis symptoms, providing direct evidence that dermal allergen exposure has the potential to exacerbate rather than ameliorate allergic disease, with implications for novel immunotherapy delivering allergen to the skin.

**Clinical implications**

Repeated intradermal allergen exposure has the potential to exacerbate rather than ameliorate allergic airway disease, with possible implications for novel immunotherapy strategies that promote dermal allergen exposure.

## Figures and Tables

**Table E1:** Verification of participant blinding

Patient guess trial arm	Trial arm	
	Control subjects (n = 43)	Subjects receiving intradermal immunotherapy (n = 44)
Intradermal immunotherapy (n = 44)	22	22
Control (n = 43)	21	22

At the end of the pollen season, participants verified blinding by guessing whether they had received active or control treatment.

**Table E2:** Effect of intradermal immunotherapy on primary and secondary outcomes (intention-to-treat analysis)

Clinical outcome	Control subjects (n = 47), median (IQR)	Intradermal immunotherapy (n = 46), median (IQR)	Difference (95% CI)	*P* value
Primary outcome				
CSMS during entire season	509 (365-738)	502 (333-841)	8 (−174.7 to 210.9)	.91
Secondary outcomes				
Symptom score during entire season	264 (156-434)	335 (183-525)	61 (−7.8 to 123.2)	.22
Medication score during entire season	263 (129-482)	242 (116-405)	−24 (−173.1 to 107.5)	.39
CSMS score during peak season	370 (292-573)	363 (232-570)	−11 (−95.8 to 77.5)	.80
Nasal symptom score during entire season	131 (80-200)	178 (120-218)	33 (0.3 to 68.5)	.03
Mouth symptom score during entire season	14 (6-45)	39 (8-90)	11 (3.1 to 26.1)	.05
Eye symptom score during entire season	78 (52-180)	79 (41-158)	−7 (−20.0 to 3.0)	.51
Lung symptom score during entire season	12 (0-40)	20 (3-32)	4 (−1.0 to 15.3)	.17
Nasal allergic symptoms measured by VAS	124 (66-166)	162 (107-275)	59 (−3.7 to 133.2)	.02
Eye allergic symptoms measured by VAS	112 (42-169)	97 (37-197)	2 (−45.6 to 49.0)	.56
Global evaluation of symptom scores	3 (1-3)	3 (2-4)	0 (0 to 1)	.43
Symptom-free days	41 (23-61)	35 (19-53)	−6 (−17 to 3)	.15
No. of days prednisone used during entire season	0 (0-0)	0 (0-0)	0 (0 to 0)	.36
Medication-free days	76 (56-94)	81 (65-93)	4 (−11.0 to 21.0)	.22
Mini-RQLQ	18 (10-25)	16 (13-23)	−0.3 (−4.2 to 3.7)	.89
EQ-5D-5L	88 (81-94)	87 (83-94)	9 (−24.8 to 43.6)	.59

Missing data were imputed. Data for the primary outcome and all symptom scores represent AUC values. Median difference between groups was calculated by using stratified Hodges-Lehmann estimation. *P* values were based on the stratified Mann-Whitney *U* (Van Elteren) test adjusted for stratification factors. *P* values for mini-RQLQ and EQ-5D-5L scores were based on a linear mixed model adjusted for stratification factors. The entire grass pollen season was from May 13-August 31, 2013; the peak season was from June 12-July 26, 2013.

*CSMS*, Combined symptom and medication score; *EQ-5D-5L*, EuroQoL instrument.

**Table E3:** Frequency of adverse events reported from first intradermal allergen immunotherapy or control injection until end of pollen season

	Control subjects (n = 47)				Subjects receiving intradermal immunotherapy (n = 46)			
	No. of participants with ≥1 AE	Percentage of participants	No. of events	Event rate (%)	No. of participants with ≥1 AE	Percentage of participants	No. of events	Event rate (%)
Any AEs	42	89	145		40	87	148	
Serious AE	2	4.3	2	1.4	1	2.2	1	0.7
Tonsillitis	0	0	0	0	1	2.2	1	0.7
Overnight stay for polysomnography	1	2.1	1	0.7	0	0	0	0
Extraction of infected dental plate	1	2.1	1	0.7	0	0	0	0
Relation of AE to treatment								
Definite/probable	6	13	14	9.7	3	6.5	15	10
Possible	0	0	0	0	0	0	0	0
Remote	34	72	70	48	30	65	68	46
None	34	72	61	42	32	70	65	44
AE withdrawals	0	0	0	0	0	0	0	0
Systemic Adverse reactions	6	13	13	9.0	3	6.5	15	10
Generalized pruritus	4	8.5	9	6.2	2	4.3	8	5.4
IgE-mediated lymphangitis	0	0	0	0	1	2.2	7	4.7
Lightheadedness	2	4.3	2	1.4	0	0	0	0
Facial flushing/feeling hot	2	4.3	3	2.1	0	0	0	0
Systemic adverse reactions∗								
Grade 1	6	13	12	8.3	3	6.5	15	10
Grade 2	0	0	0	0	0	0	0	0
Grade 3	0	0	0	0	0	0	0	0
Grade 4	0	0	0	0	0	0	0	0

Statistical comparison was done by using the Fisher exact test for 5 or fewer events and the χ^2^ test for more than 5 events.

*AE*, Adverse event.

∗ Classified by using the World Allergy Organization grading system for systemic reactions to subcutaneous immunotherapy.^E3^

**Table E4:** Microarray gene expression profiles of activated CD4^+^ T cells derived from skin biopsy explants

Gene	*P* value	Fold difference
Intradermal immunotherapy down vs control group		
*LOC100133042*	.02	−1.80
*CEP55*	.03	−1.78
*GFOD1*	.00	−1.77
*HIST2H2AB*	.04	−1.62
*H2AFZ*	.02	−1.61
*LOC730534*	.01	−1.57
*HSD17B4*	.02	−1.57
*HIST1H2AD*	.03	−1.56
*HDAC1*	.01	−1.55
*CCL3L1*	.03	−1.53
*CALR*	.02	−1.52
*CDCA5*	.01	−1.52
*PRDX5*	.01	−1.51
*FEN1*	.02	−1.50
Intradermal immunotherapy up vs control group		
*EPS15*	.02	1.51
*MYB*	.01	1.52
*GK*	.03	1.53
*RNASET2*	.03	1.55
*LOC729383*	.02	1.56
*GPR171*	.00	1.59
*LOC729387*	.04	1.60
*SLC11A2*	.02	1.60
*HS.508682*	.04	1.68
*IL5*	.03	1.71
*GBP5*	.05	1.79
*TNFSF8*	.01	1.79
*TNIP3*	.03	1.87
*CENTA1*	.05	2.11

Data were analyzed by using the 3 way-ANOVA model.

## Methods

### Participants

Full inclusion criteria were adults aged 18 to 65 years with a history of moderate-to-severe symptoms of grass pollen–induced allergic rhinitis according to ARIA classification^E1^ in May, June, or July for a minimum of 2 years, interfering with daily activities or sleep and remaining troublesome despite treatment with medication. Participants were required to have a positive skin prick test response (wheal diameter ≥3 mm) to *P pratense* together with a positive specific IgE level (IgE class 2 or greater) against *P pratense*. Women of childbearing age were included if they were willing to use an effective form of contraception for the duration of intradermal injections. Participants were able to consent and comply with study procedures.

Exclusion criteria were as follows: prebronchodilator FEV_1_ less than 70% of predicted value at screening; seasonal grass pollen–induced asthma requiring regular treatment with albuterol or inhaled corticosteroids (those with mild seasonal grass pollen–induced asthma controlled with occasional albuterol only were included); significant symptomatic seasonal allergic rhinitis, asthma, or both caused by tree or weed pollen near or overlapping the grass pollen season (patients with mild intermittent symptoms requiring only occasional antihistamines were included); significant perennial rhinitis (patients with mild intermittent symptoms requiring only occasional antihistamines were included); an emergency department visit for asthma in the previous 12 months; chronic obstructive pulmonary disease; recurrent acute sinusitis; chronic sinusitis; previous grass pollen immunotherapy within the previous 5 years; previous life-threatening anaphylaxis or angioedema; history of intolerance of grass pollen immunotherapy or rescue medications; a positive serum or urine pregnancy test result within 72 hours of enrollment; lactating women; use of any investigational or immunosuppressive drug within 30 days of screening; use of leukotriene receptor antagonists, β-blockers, calcium-channel blockers, tricyclic antidepressants, monoamine oxidase inhibitors, or anti-IgE mAb; a medical condition that the investigator deemed incompatible with participation in the trial; infection of the upper respiratory tract, sinuses, or middle ear at randomization; and insufficient understanding of the trial protocol. Current smokers or subjects with greater than or equal to 5 pack years of smoking were also ineligible.

### Power calculations

Sample size calculations for the primary outcome (combined symptom and medication score) were performed based on raw data from a previous clinical trial of subcutaneous grass pollen immunotherapy.^E2^ The power calculation was conservatively based on the detection of a clinical effect size 80% of that reported in that trial. By using this method and a 2-sided nonparametric test based on a Monte Carlo approach, group sample sizes of 35 and 35 achieved 90% power to detect such a difference in AUC of the combined symptom and medication scores at a significance level of .05. The sample size was increased by a further 15% to 40 in each arm to make allowance for the unknown distribution of the primary outcome and based on the lower bound for the asymptotic relative efficiency of the Mann-Whitney *U* test. Further accounting for a postrandomization dropout rate of up to 10%, which is consistent with previous trials of grass pollen immunotherapy, a total sample size of 90 (45 each arm) was estimated as required.

### Skin biopsy randomization

In August 2013, the King's Clinical Trial Unit randomly selected participants to be approached to undergo skin biopsies. The first 40 participants who consented then underwent biopsy. Also in August 2013, all participants were randomized a second time to one of 3 groups for repeat intradermal allergen injections at 7, 10, or 13 months after the final intradermal immunotherapy or control injection to assess whether low-dose intradermal allergen immunotherapy was associated with prolonged suppression of skin responses.

### Masking

All physicians, researchers, research nurses, outcome assessors, and patients were blinded to treatment allocation until primary and secondary analyses were complete. Active and control study medication vials appeared identical. Only the King's Clinical Trial Unit randomization provider and the manufacturing pharmacy had access to blinding information. Unmasking could be performed for emergencies only. To verify blinding, participants guessed whether they had received the active or control intervention after the pollen season.

### Procedures

Each active intradermal allergen injection contained 10 BU (33.3 SQ-U) of *P pratense* soluble grass pollen extract (Aquagen SQ Timothy; ALK-Abelló) in a 20-μL volume (ie, 500 BU/mL [1666.7 SQ-U/mL]). Individual vials for each participant and each visit were preprepared and prelabeled by Guy's Hospital Pharmacy under good manufacturing practice conditions. In brief, Aquagen SQ Timothy grass pollen extract was reconstituted in manufacturer-supplied diluent to the maximum recommended concentration (30,000 BU/mL [100,000 SQ-U/mL] ie, 60× final working strength; shelf life of 6 months at 2°C to 8°C after reconstitution), and 0.15 mL was placed in aliquots into glass study vials. At each visit, for intradermal injection, the investigator added 8.85 mL of clinical grade 0.9% normal saline at ambient temperature to the vial corresponding to that participant's visit to achieve a 60-fold dilution. Twenty microliters was then aspirated from this vial and administered directly. The allergen required dilution on the day of administration because the recommended shelf life of Aquagen SQ Timothy Grass Pollen extract at 500 BU/ml (1666.7 SQ-U/mL) is 14 days. The control intervention was histamine only administered at a concentration of 100 μg/mL for the first and second injections. Histamine concentrations were reduced to 30 μg/mL for the third and fourth injections and 10 μg/mL for the fifth, sixth, and seventh injections to help preserve blinding. Histamine was also placed in aliquots in study vials at 60× final working strength in 0.15-mL volumes for dilution with 8.85 mL of clinical grade 0.9% normal saline immediately before injection to match the grass pollen extract dilution and preserve blinding. Active and control study medications appeared identical.

The injection site was alternated between the left and right arms at each visit. Intradermal injections were administered in a 20-μL volume by using a 29-gauge insulin syringe (Micro-Fine; BD Biosciences, Oxford, United Kingdom). In the event of an injection being administered too deeply (ie, into subcutaneous tissue) to elicit an immediate injection “bleb” and subsequent characteristic wheal, the injection was repeated 1 cm from the original site. After an intradermal injection, participants were able to take an antihistamine to reduce the local itching and swelling, if they wished.

### Study outcomes

The primary outcome was a combined symptom and medication score during the grass pollen season (May 13-August 31, 2013; 111 days), as recommended by WAO guidelines for allergic rhinitis immunotherapy trials.^E3^ Participants scored symptoms from 0 to 3 in the nose (sneezing, blockage, and running), eye (itching, redness, tears, and swelling), mouth, and throat (itching and dryness), and chest (breathlessness, cough, wheezing, and tightness). Daily rescue medication was scored as follows: desloratadine, 5 mg, up to 1 tablet daily (6 points daily); olopatadine eye drops, 1 mg/mL, up to 1 drop per eye twice daily (1.5 points per drop; maximum, 6 points daily); fluticasone nasal spray, 50 μg per spray, up to 2 sprays per nostril once daily (2 points per spray; maximum, 8 points daily); and prednisone, 5 mg per tablet, up to 6 tablets daily (2 points per tablet; maximum, 12 points daily). Symptom and medication scores were expressed as the AUC for the entire grass pollen season. Scores for symptoms (maximum, 39 points daily) and medications (maximum, 32 points daily) were normalized before combining, as recommended by the WAO.^E3^

### Safety

#### Adverse events

Adverse events and side effects were recorded from the first treatment injection throughout the study regardless of severity or relation to study participation. As a precaution against systemic allergic reactions, all participants were observed after the first injection for 1 hour, and if there was no systemic reaction, they were observed for 30 minutes after subsequent injections. In the event of experiencing a grade 1 reaction, the observation period for that subject remained at 1 hour after subsequent injections.

The following adverse events were anticipated and not reported:

1. symptoms caused by aeroallergen exposure (ie, nasal blockage, rhinorrhea, itching, or sneezing; itching, watering, redness, or swelling of the eyes; itching or dryness of the mouth/throat; or breathless, cough, wheeze, and chest tightness);
2. transient discomfort from intradermal injections;
3. appearance of an itchy edematous wheal with surrounding erythema after intradermal injection;
4. appearance of swelling (edema) within hours of intradermal injection;
5. temporary discomfort, bleeding, bruising, and swelling at the needle site after venesection; and
6. mild localized itching arising from skin prick testing during screening.

#### Withdrawal criteria and stopping rules

The prespecified criteria for discontinuation of the study therapy (active or control) were as follows:

1. inability or failure to attend intervention within 3 weeks of previous allergen/histamine administration;
2. inability or failure to receive 7 or 8 injections within the dates specified;
3. 2 grade 2 systemic reactions or a single systemic reaction of grade 3 or greater after administration of study therapy, with systemic reactions graded according to the WAO criteria^E4^:
  A. *Grade 1*: symptoms of 1 organ system (cutaneous, upper respiratory tract, conjunctival, gastrointestinal, and other);
  B. *Grade 2*: symptoms of more than 1 organ system present or asthma symptoms/signs (cough, wheezing, and shortness of breath but <40% decrease in peak expiratory flow or FEV_1_);
  C. *Grade 3*: asthma symptoms/signs (with ≥40% decrease in peak expiratory flow or FEV_1_), upper respiratory tract (laryngeal, uvula, and tongue) edema with or without stridor; or
  D. *Grade 4*: respiratory failure or hypotension with or without loss of consciousness;
4. an adverse event that, in the judgment of the principal investigator or the medical monitor, presented an unacceptable consequence or risk to the participant;
5. an illness or infection not associated with the condition under study and that required treatment not consistent with protocol requirements or if a participant had an intercurrent illness that, in the judgment of the principal investigator, in any way justified discontinuation;
6. an inability or unwillingness to comply with the study protocol, with the protocol deviations being sufficient to jeopardize the participant's well-being or the integrity of the study; and
7. pregnancy occurring during study participation.

Predefined study stopping rules included the occurrence of 5 grade 3 reactions or a single grade 4 reaction.

### Intradermal skin challenge testing

All patients underwent intradermal skin challenge testing 4 months after the final intradermal allergen immunotherapy or control injection (September 2013). Participants were then randomized to undergo a repeat follow-up test at either 7, 10, or 13 months to assess persistence of late response suppression by comparing late-phase response sizes in those who had received active intradermal immunotherapy or the control intervention. The procedure for the intradermal skin challenge testing and the dose of allergen used was identical to that for an active intradermal allergen immunotherapy injection. In brief, grass pollen extract (10 BU, equivalent to 33.3 SQ-U, of Aquagen SQ Timothy; ALK-Abelló) in a 20-μL volume of allergen diluent was injected intradermally into the extensor aspect of each forearm. A negative control injection of 20 μL of diluent was injected into the contralateral forearm. Participants were asked to refrain from taking antihistamines or oral steroids for a minimum of 5 days and 2 weeks beforehand, respectively. Early-phase responses were measured 15 minutes after the intradermal injection. The wheal outline was traced and transferred into the patient record. Late-phase responses were measured after 24 hours by means of palpation of the outline of edema. The area of the late response was also traced and transferred to the patient record. A single clinician performed all measurements under double-blind conditions. The early- and late-phase response areas were calculated from scaled scanned images of the tracings with NIS Elements v4.2 software (Nikon Instruments, Tokyo, Japan). Early- and late-phase response areas were then compared in the intradermal immunotherapy and control arms at each time point.

### Skin biopsy

Forty participants (20 in each trial arm) were randomized to undergo 3-mm skin punch biopsies immediately after measurement of late-phase responses (ie, 24 hours after challenge) 4 months after completing their final treatment injections in September 2013. Biopsy specimens were collected from both allergen-challenged and diluent control sites. Local anesthesia was achieved with 10 mg/mL lidocaine hydrochloride with adrenaline 1/200,000 (5 μg/mL). In the first 20 subjects, biopsy specimens were divided with a scalpel into 2 pieces, and one-half piece was fixed in 4% paraformaldehyde (Sigma-Aldrich, Poole, United Kingdom) for 2 hours. In the rest of the subjects, entire biopsy specimens were processed for immunohistochemistry by means of fixation in 4% paraformaldehyde at room temperature for 4 hours. After washing twice in 15% sucrose, biopsy specimens were mounted in OCT embedding medium (Bayer UK, Basingstoke, United Kingdom) and stored at −80°C pending analysis. The remaining unfixed half-biopsy pieces were cultured directly for T-cell analysis.

### Analysis of T cells cultured from skin biopsy specimens

Skin biopsy tissue was finely dissected and resuspended in complete medium (RPMI supplemented with 10% FCS, penicillin [100 U/mL], streptomycin [100 μg/mL], and L-glutamine (2 mmol/L; all from Life Technologies, Warrington, United Kingdom). Tissues were then cultured at 37°C in a humidified atmosphere containing 5% CO_2_ in the presence of IL-2 (50 U/mL). After 3 to 4 days, cells were passed through a 0.2-μm cell strainer to obtain single-cell suspensions and restimulated with immobilized anti-CD3/CD28 antibodies for a further 3 days, followed by expansion for 4 days in the presence of IL-2.

Expanded T cells were stained with the viability dye eFluor 780 (eBioscience, Vienna, Austria) before surface staining with anti-CD4 peridinin-chlorophyll-protein complex–Cy5.5 (BioLegend, London, United Kingdom), anti-CD8 Brilliant Violet 510 (BD Biosciences), anti-CRTH2 phycoerythrin (PE; BioLegend), anti-CXCR3 Brilliant Violet 421 (BioLegend), anti-CCR6 PE-Cy7 (BD Biosciences), and anti-IL-25 receptor Alexa Fluor 647 (a kind gift of Dr Andrew McKenzie). Samples were resuspended (FACSFlow, BD Biosciences) for flow cytometric analysis (FACSCalibur, BD Biosciences). Data were analyzed with FlowJo v7.6 software (TreeStar, Ashland, Ore).

For microarray studies, cells were activated for 4 hours with ionomycin (500 ng/mL) and phorbol 12-myristate 13-acetate (5 ng/mL; both from Sigma-Aldrich). RNA was isolated from cell pellets by using the miRNeasy mini kit and RNeasy MinElute cleanup kit (Qiagen, Manchester, United Kingdom), according to the manufacturer's instructions. cDNA synthesis and amplification were performed with the Ovation PicoSL WTA system V2 kit (NuGEN, Leek, The Netherlands) per the manufacturer's instructions. Purity and yield were then analyzed by using the Bioanalyzer platform (Agilent, Stockport, United Kingdom) and the NanoDrop 2000 spectrophotometer (Thermo Scientific, Loughborough, United Kingdom), respectively, before amplified cDNA was biotin labeled with the NuGEN Encore BiotinIL Module, according to the manufacturer's instructions. Biotin-labeled cDNA was hybridized to an Illumina Human HT-12 v4 Expression BeadChip before scanning with the iScan system (Illumina, Essex, United Kingdom) with GenomeStudio software. Data analysis was performed with the Partek Genomics Suit software (Partek, St Louis, Mo).∗

### Immunohistochemistry

Immunohistochemical staining of skin biopsy specimens was performed with the modified alkaline phosphatase–anti-alkaline phosphatase (APAAP) method to stain for eosinophils, neutrophils, CD4^+^ T cells, and CD3^+^ T cells.E5, E6 In brief, 8- to 10-μm thickness tissue sections were air-dried overnight on poly-L-lysine–coated slides. For immunostaining, slides were incubated at room temperature in a humidified chamber with the primary mouse mAb (neutrophil elastase; Dako, Ely, United Kingdom; eosinophil major basic protein; Abcam, Cambridge, United Kingdom; or CD3 and CD4, both from Dako) suspended in 5% human serum/PBS for predetermined optimized incubation times. Sections were then washed in PBS and incubated with rabbit anti-mouse immunoglobulin (Dako) for 30 minutes and then washed again. Slides were then incubated with a third layer of soluble complexes of AP and mouse anti-APAAP (Serotec, Kidlington, United Kingdom) for 30 minutes, washed, and developed with Fast Red (Sigma-Aldrich) for a further 20 minutes. Sections were washed extensively in PBS before counterstaining with Harris' hematoxylin (BDH, Poole, United Kingdom) and mounted in glycerol gel. For negative controls, each primary antibody was substituted with the appropriate isotype-matched irrelevant mAb. Slides were counted blind in random order by 2 observers. Allergen and diluent biopsy sections were evaluated from each subject. The total number of positive cells was expressed as the number of cells per square millimeter of biopsy specimen. Interobserver variability was 7%, as assessed on repeat counts of 19 slides. The difference between the 2 counts was plotted against the mean of the 2 counts; all but one of the differences fell within 2 SDs of the mean difference, indicating satisfactory agreement between observers.

### Serum antibody measurements

Sera were analyzed for concentrations of *P pratense*–specific IgG, IgG_4_, and IgE and IgE specific to the major allergens Phl p 5 and Phl p 1 by using a commercial assay system, according to the manufacturer's instructions (ImmunoCAP; Thermo Fisher Scientific, Horsham United Kingdom).

### Basophil activation test

Basophil activation tests were performed in 92 participants after administration of the final intradermal allergen immunotherapy or control injection (May 2013). Whole blood was collected and tested within 2 hours of sampling under blinded conditions by a single investigator (A.G.). Heparinized whole blood was immunostained with anti-human CD3 PE-Cy7 (BD Biosciences), CD294 PE (Miltenyi Biotec, Woking, United Kingdom), CD203c peridinin-chlorophyll-protein complex–Cy5.5 (BioLegend), CD303 allophycocyanin (Miltenyi Biotec), CD107a Brilliant Violet 421 (BioLegend), CD63 FITC (BioLegend), and isotype controls. Basophils were then stimulated with anti-human IgE (1000 ng/mL, positive control; Abcam) or *P pratense* extract (ALK-Abelló) at 10 and 100 ng/mL for 15 minutes at 37°C. Samples were then lysed (BD FACS Lysing Solution, BD Biosciences), washed, and resuspended (CellFix, BD Biosciences) for flow cytometric analysis (FACSCalibur, BD Biosciences). Data were analyzed with FlowJo v7.6 software (TreeStar), gating on CD3^−^CD303^−^CD294^+^ basophils. Basophil activation was expression as the percentage CD63^+^, CD203c^+^, or CD107a^+^ basophils of the entire basophil population.
